# In Vitro Antimicrobial Activity of Volatile Compounds from the Lichen *Pseudevernia furfuracea* (L.) Zopf. Against Multidrug-Resistant Bacteria and Fish Pathogens

**DOI:** 10.3390/microorganisms12112336

**Published:** 2024-11-15

**Authors:** Yasser Essadki, Adel Hilmi, Antonio Cascajosa-Lira, Mariana Girão, El Mehdi Darrag, Rosário Martins, Abderrahmane Romane, Soukaina El Amrani Zerrifi, Richard Mugani, Zakaria Tazart, El Mahdi Redouane, Angeles Jos, Ana M. Cameán, Vitor Vasconcelos, Alexandre Campos, Fatima El Khalloufi, Brahim Oudra, Mustapha Barakate, Maria de Fátima Carvalho

**Affiliations:** 1 Water, Biodiversity and Climate Change Laboratory, Phycology, Biotechnology and Environmental Toxicology Research Unit, Faculty of Sciences Semlalia Marrakech, Cadi Ayyad University, Bd Prince Moulay Abdellah, Marrakech 40000, Morocco; yasser.essadki@ced.uca.ma (Y.E.); soukainaelamranizerrifi@gmail.com (S.E.A.Z.); richardmugani@gmail.com (R.M.); oudra@uca.ac.ma (B.O.); 2 Laboratory of Microbial Biotechnology, AgroSciences and Environment, CNRST Labeled Research Unit N◦4, Faculty of Sciences Semlalia, Cadi Ayyad University, Bd Prince Moulay Abdellah, Marrakech 40000, Morocco; hilmiadel87@gmail.com (A.H.); mbarakate@uca.ma (M.B.); 3 Área de Toxicología, Facultad de Farmacia, Universidad de Sevilla, C. Tramontana, 2, 41012 Sevilla, Spain; aclira@us.es (A.C.-L.); angelesjos@us.es (A.J.); camean@us.es (A.M.C.); 4 Interdisciplinary Centre of Marine and Environmental Research (CIIMAR/CIMAR), University of Porto, Terminal de Cruzeiros do Porto de Leixões, 4450-208 Matosinhos, Portugal; mariana.martins@ciimar.up.pt (M.G.); mrm@ess.ipp.pt (R.M.); vmvascon@fc.up.pt (V.V.); amoclclix@gmail.com (A.C.); 5 Laboratory of Applied Chemistry and Biomass, Faculty of Sciences Semlalia Marrakech, Cadi Ayyad University, Bd Prince Moulay Abdellah, Marrakech 40000, Morocco; elmehdi.darrag@gmail.com (E.M.D.); a.romane@gmail.com (A.R.); 6 Health and Environment Research Centre, School of Health, Polytechnic Institute of Porto (E2S/P.PORTO), R. Dr. António Bernardino de Almeida 400, 4200-072 Porto, Portugal; 7 Higher Institute of Nurses Professions and Health Techniques of Guelmim, Guelmim 81000, Morocco; 8 AgroBioSciences Department, College of Agriculture and Environmental Sciences, Mohammed VI Polytechnic University, Ben Guerir 43150, Morocco; zakaria.tazart@um6p.ma; 9 UMR-I 02 INERIS-URCA-ULH SEBIO, Stress Environnementaux et BIOsurveillance des Milieux Aquatiques, Université de Reims Champagne-Ardenne, Campus du Moulin de la Housse, BP 1039, 51687 Reims, CEDEX, France; el-mahdi.redouane@univ-reims.fr; 10 Department of Biology, Faculty of Sciences, University of Porto (FCUP), Rua Campo Alegre s/n, 4169-007 Porto, Portugal; 11 Natural Resources Engineering and Environmental Impacts Team, Multidisciplinary Research and Innovation Laboratory, Polydisciplinary Faculty of Khouribga, Sultan Moulay Slimane University of Beni Mellal, Bd 2 Mars, Khouribga 25000, Morocco; elkhalloufi.f@gmail.com; 12 ICBAS—School of Medicine and Biomedical Sciences, University of Porto, R. Jorge de Viterbo Ferreira 228, 4050-313 Porto, Portugal

**Keywords:** lichens, *Pseudevernia furfuracea*, volatile compounds, multidrug-resistant bacteria, fish pathogens, minimum inhibitory concentration

## Abstract

Lichens are symbiotic organisms with unique secondary metabolism. Various metabolites from lichens have shown antimicrobial activity. Nevertheless, very few studies have investigated the antimicrobial potential of the volatile compounds they produce. This study investigates the chemical composition and antimicrobial properties of volatile compounds from *Pseudevernia furfuracea* collected in two regions of Morocco. Hydrodistillation was used to obtain volatile compounds from samples collected in the High Atlas and Middle Atlas. Gas chromatography–mass spectrometry (GC-MS) analysis identified phenolic cyclic compounds as the primary constituents, with atraric acid and chloroatranol being the most abundant. Additionally, eight compounds were detected in lichens for the first time. The antimicrobial activity of these compounds was assessed using disc diffusion and broth microdilution methods. Both samples demonstrated significant antimicrobial effects against multidrug-resistant human bacteria, reference microorganisms, fish pathogens, and *Candida albicans*, with minimum inhibitory concentrations (MICs) ranging from 1000 µg/mL to 31.25 µg/mL. This study provides the first report on the volatile compounds from *Pseudevernia furfuracea* and their antimicrobial effects, particularly against fish pathogens, suggesting their potential as novel antimicrobial agents for human and veterinary use. Further research is warranted to explore these findings in more detail.

## 1. Introduction

Lichenization is a nutritional specialization of lichen-forming fungi [1], wherein the exhabitant fungus and inhabitant autotrophic algae or cyanobacteria interact for mutual benefit, forming a symbiotic association [2]. The morphology of the lichen thallus is highly diverse, ranging from leprose to fruticose [3]. Approximately 20,000 species of lichens have been described worldwide [4]. They produce around one thousand secondary metabolites, primarily through the shikimic acid, mevalonic acid, and polymalonate pathways [5]. These metabolites have demonstrated antioxidant, anticancer, and antimicrobial potential [6]. For instance, usnic acid, one of the most studied lichen metabolites, has exhibited antibacterial activity against *Staphylococcus aureus* and *Bacillus subtilis*, inhibiting DNA and RNA synthesis [7]. 

In Morocco, a catalogue updated in 2013 stated the presence of 1169 lichen species, distributed in 21 orders, 75 families, and 241 genera [8]. A more recent revision was provided based on the changes in nomenclature and indicated that Moroccan national biodiversity includes 1237 lichen species [9]. Amidst this broad national diversity, *Pseudevernia furfuracea* (L.) Zopf, a lichen of the Parmeliaceae family, is found in large populations in Morocco’s Middle and High Atlas regions [10], and in North Africa in general [11]. This lichen species was widely used in the fragrance industry under the appellation ‘cedarmoss’ when growing on *Cedrus atlantica*, or ‘treemoss’ when growing on other trees [12]. In 1997, 1900 tons of this lichen, harvested globally, including in Morocco, were processed in France [12]. In Morocco, it has been traditionally used as a decoction believed to improve blood volume and aid in liver detoxification [13]. While the antimicrobial potential of extracts from this lichen has been investigated worldwide and in Morocco [14,15,16], to our knowledge, no research has been conducted on the antimicrobial potential of its volatile compounds.

With an extensive diversity of secondary metabolites exhibiting antimicrobial properties, lichens, such as *Pseudevernia furfuracea*, present promising natural alternatives to synthetic antibiotics. These compounds could play an important role in addressing antimicrobial resistance (AMR), a challenge of growing concern to human health. Indeed, the World Health Organization (WHO) identifies AMR as one of the top three threats to public health [17]. It occurs when pathogens—bacteria, fungi, parasites, and viruses—stop responding to compounds that previously inhibited them [18]. This resistance complicates the treatment of infections or even renders it ineffective. In 2019 alone, AMR was estimated to contribute to 1.27 million deaths, and predictive models suggest that this number could reach 10 million by 2050 [19]. Among the most concerning “superbugs” are methicillin-resistant *Staphylococcus aureus* (MRSA), multidrug-resistant *Acinetobacter baumannii*, carbapenem-resistant *Klebsiella pneumoniae*, and strains of *Escherichia coli* [19,20]. Additionally, *Mycobacterium tuberculosis* remains a global health threat, especially in developing countries [19]. Beyond the death toll, AMR also poses a significant economic burden, potentially costing trillions of dollars worldwide [17]. The One Health approach, which emphasizes the connections between human, animal, and environmental health [18], highlights the link between human health and aquaculture. Indeed, the field of aquaculture has experienced a significant rise over the past 40 years, driven by global demographic growth and the need to provide a sustainable protein source [21,22,23]. By 2017, the annual production of fish and shellfish reached 80 million tons, with freshwater species accounting for approximately 45 million tons and Asia contributing over 97% of global production [21]. Commonly farmed freshwater species include grass carp, silver carp, Nile tilapia, and common carp [21]. However, the rapid expansion of aquaculture has introduced serious challenges, such as the proliferation of pests, parasites, and pathogens (PPP), pollution, harmful algal blooms, and climate change [21]. To control infections and maintain production yields, antibiotics are often used in aquaculture, not only to treat disease but also as growth promoters [24,25]. This widespread use of antibiotics in aquaculture has consequences that reach far beyond the industry itself, as these compounds can persist in water systems and promote the development of AMR in both aquatic and human pathogens [26]. Resistant bacteria and resistance genes can spread through waterways, potentially reaching human populations and contributing to the global AMR crisis [26,27]. As such, the reliance on antibiotics in aquaculture intensifies the AMR issue in humans, making it imperative to find alternative antimicrobial agents for sustainable aquaculture practices [28]. Among alternative strategies, aromatic and medicinal plants have shown promise in combatting fish pathogens while reducing reliance on traditional antibiotics [29,30]. Essential oils, for instance, contain bioactive volatile compounds that are effective against aquaculture pathogens. Eugenol-rich oil from *Syzygium aromaticum* and limonene-rich oil from *Citrus limon*, as well as thymol-rich oil from *Thymus vulgaris*, have demonstrated strong antibacterial activity against various fish pathogens in vitro [31,32]. These findings underscore the potential of herbal compounds not only in aquaculture but also in medical applications as eco-friendly alternatives to synthetic antibiotics. Therefore, this work aimed to investigate for the first time the chemical composition and antimicrobial potential of volatile compounds from *P. furfuracea*, harvested from two different locations in Morocco. Antimicrobial activity was screened against a wide range of microorganisms that included reference bacterial strains, multidrug-resistant clinical human isolates, fish pathogenic bacteria, and a pathogenic yeast.

## 2. Materials and Methods

### 2.1. Sample Collection, Volatile Compound Extraction, and Chemical Characterization

#### 2.1.1. Sample Collection

Lichens (Figure S1) were collected in June 2021 from two different locations in Morocco, one on *Quercus rotundifolia* L. near Marrakech in the High Atlas region (31°28′19.5″ N 7°25′27.6″ W, 1644 m), and the other on *Cedrus atlantica* (Manetti ex Endl.) Carriere near Azrou in the Middle Atlas region (33°25′2″ N 5°11′10″ W, 1776 m). Biomass was harvested manually from the low branches of the different substrates. Lichen identification was conducted by one of the authors (Y.E.) using determination keys [33] and online resources [34]. The results were as follows: Medulla C−, K+, and cortex C−, K− for both samples. The lichens were identified as *Pseudevernia furfuracea* var. *furfuracea*. Voucher specimens were deposited in the personal herbarium of one of the authors (Y.E.) under the reference numbers 9 and 15.

#### 2.1.2. Volatile Compound Extraction

Volatile compound extraction was performed according to the protocol of Sanad et al. [35]. Briefly, the collected biomass was stored under shaded conditions at room temperature (±25 °C) on a laboratory bench for one week to eliminate excess moisture. Manual sorting was then executed to eliminate contaminants such as branches, bark, debris, and other lichens. Following the drying and sorting processes, the biomass was ground using an electrical grinder. Subsequently, 100 g of the powdered lichens was mixed in a hydrodistillation apparatus with 4 L of deionized water. Distillation was conducted for a period of 4 h. The obtained distillate was then mixed thoroughly with dichloromethane in a separatory funnel. Following complete phase separation (with two clear phases visible in the funnel), the dichloromethane phase was collected and evaporated in a rotary evaporator at 30 °C. The obtained volatile compounds were therefore stored in amber glass vials at 4 °C. *Pseudevernia furfuracea* volatile compounds (PFVCs) from the High Atlas region were attributed the code 1, and PFVCs from the Middle Atlas region the code 2.

#### 2.1.3. Gas Chromatography–Mass Spectrometry Protocol and Molecular Characterization

The chromatographic separation and subsequent identification of compounds were carried out using a gas chromatograph coupled to a quadrupole-Orbitrap hybrid mass spectrometer (GC-MS) QExactive (ThermoFisher Scientific, Waltham, MA, USA) equipped with an electron impact ionization source operating in positive mode. Xcalibur 4.3 software (ThermoFisher Scientific, Waltham, MA, USA) was employed for instrument control and data acquisition, while Trace Finder 5.1 software (ThermoFisher Scientific, Waltham, MA, USA) was used for result processing. GC analysis was conducted using an Agilent Technologies chromatographic column (30 m × 0.25 mm, 0.25 µm particle size). Helium was utilized as the carrier gas. The temperature ramp protocol consisted of an initial isothermal hold at 60 °C for 1 min, followed by a gradient increase of 5 °C/min up to 210 °C, then of 10 °C/min up to 280 °C, and finally, the temperature was maintained for 15 min. The injection volume was 1 µL, and the analysis was performed in Split mode with a Split ratio of 1:50. The injector temperature was set at 250 °C, and the carrier gas flow was held constant at 1 mL/min. Electron impact ionization was set to 70 eV, employing a Full Scan analysis method covering the *m*/*z* range of 35–650 in profile mode with a resolution of 60,000.

To determine the composition of both extracts, Compound Discoverer™ 3.2 software (Thermo Fisher Scientific, Waltham, MA, USA) was used. The GC-EI Deconvolution node was employed to differentiate and identify overlapping compounds by selecting the appropriate library (National Institute of Standards and Technology, NIST) for molecular matching. For compounds lacking chromatographic peaks, the workflow imputes area values, yielding a comprehensive dataset. Retention Index information was also included to improve identification accuracy. After processing the GC data, a differential analysis was conducted to compare variations in compound abundance between the different extracts, facilitating a clearer understanding of compositional differences. Finally, a Molecular Network visualization was generated to illustrate potential structural relationships among compounds, offering insights into chemical families or pathways within the extracts. This combined approach provides an in-depth analysis of extract composition, enabling both the detection and structural interpretation of its constituents.

### 2.2. Antimicrobial Activity

#### 2.2.1. Reference Strains

The antimicrobial activity of PFVC1 and 2 was assessed against a group of five reference microorganisms: two Gram-positive strains (*Staphylococcus aureus* (ATCC 29213) and *Bacillus subtilis* (ATCC 6633)), two Gram-negative strains (*Salmonella typhimurium* (ATCC 25241) and *Escherichia coli* (ATCC 25922)), and one yeast (*Candida albicans* (ATCC 10231)). *S. aureus*, *B. subtilis*, *S. typhimurium*, and *E. coli* were grown on Mueller–Hinton agar (MH) (Biokar, Beauvais, France). *C. albicans* was grown on Sabouraud-Dextrose agar (SDA) (Biokar, Beauvais, France). Additional information on the culture conditions can be found in the Supplementary Materials (Table S1).

#### 2.2.2. Clinical Strains

Four additional isolates from the Intensive Care Unit of the University Hospital Mohammed VI of Marrakesh (Morocco) were also used [36]. Those isolates originated from pus, blood, tracheal aspirate, and catheters and were identified as *E. coli* (E1), *Klebsiella pneumoniae* (K1), *Acinetobacter baumannii* (A1), and *S. aureus* (S1), respectively. Identification involved a comprehensive assessment of morphological, biochemical, and cultural characteristics, supported by the API library (Biomerieux, Marcy-l'Etoile, France). Antibiotic susceptibility testing was executed according to the guidelines recommended by the Antibiogram Committee of the French Microbiology Society [37] and the European Committee on Antimicrobial Susceptibility Testing EUCAST [38]. The resistance profiles of the individual strains are provided in the Supplementary Materials (Table S2). These strains were considered multidrug-resistant as per the criteria established by Magiorakos et al. [39] and were grown on Mueller–Hinton agar (MH) (Biokar, Beauvais, France) at 37 °C for 24 h.

#### 2.2.3. Fish Pathogenic Strains

In addition, a group of 5 fish pathogenic strains were used: *Aeromonas hydrophila* (DSM 30187), *Pseudomonas anguiliseptica* (DSM 12111), *Edwardsiella tarda* (DSM 30052), *Listonella anguillarum* (ATCC 19264), and *Yersinia ruckeri* (ATCC 29473). These strains were grown on Tryptic Soy agar (TSA) (Biokar, Beauvais, France). The specific culture conditions are available in the Supplementary Materials (Table S1).

#### 2.2.4. Disc Diffusion Assay

The disc diffusion assay was conducted according to Girão et al. [40]. Briefly, microorganism suspensions in the appropriate liquid medium were prepared and adjusted to 0.5 McFarland. Sterile cotton swabs were used to inoculate the suspensions on agar plates (MH, TSA, or SDA, depending on the microorganism). Blank 6 mm Whatman paper discs were placed on the surface of the inoculated agar plates, and 10 µL of PFVCs (24 mg/mL) were loaded onto the discs. Positive control discs were loaded with Enrofloxacin (1 mg/mL) for the reference bacterial strains and with Nystatin (1 mg/mL) for the yeast. Negative control discs were loaded with Dimethylsulfoxide (DMSO). The experiment was repeated three independent times. Antimicrobial activity was determined by measuring the inhibition zone diameter (IZD) formed around the discs after the appropriate incubation time and at the appropriate temperature for each microorganism (Table S1).

#### 2.2.5. Minimum Inhibitory Concentration

The minimum inhibitory concentration (MIC) was determined using the broth microdilution assay according to the CLSI guidelines M07-A10 for bacteria [41] and M27-A3 for yeasts [42]. Briefly, a PFVC stock solution was prepared in the appropriate liquid medium at a concentration of 2000 µg/mL with 2% DMSO. Two-fold dilutions were subsequently prepared from this stock, and 100 µL of each dilution was added to microwells previously inoculated with 100 µL of the microbial inoculum (prepared by diluting the 0.5 McFarland microorganism suspension fifty-fold). The positive growth control consisted of 100 µL of microbial inoculum and 100 µL of medium broth, and the negative growth control consisted of 200 µL of medium broth. The MIC was defined as the lowest concentration of PFVC that inhibited the macroscopic growth of the tested strains. For each extract, the MIC was determined in triplicate, and two independent experiments were performed.

## 3. Results

### 3.1. Chemical Composition of the Volatile Compounds

**Figure 1:**
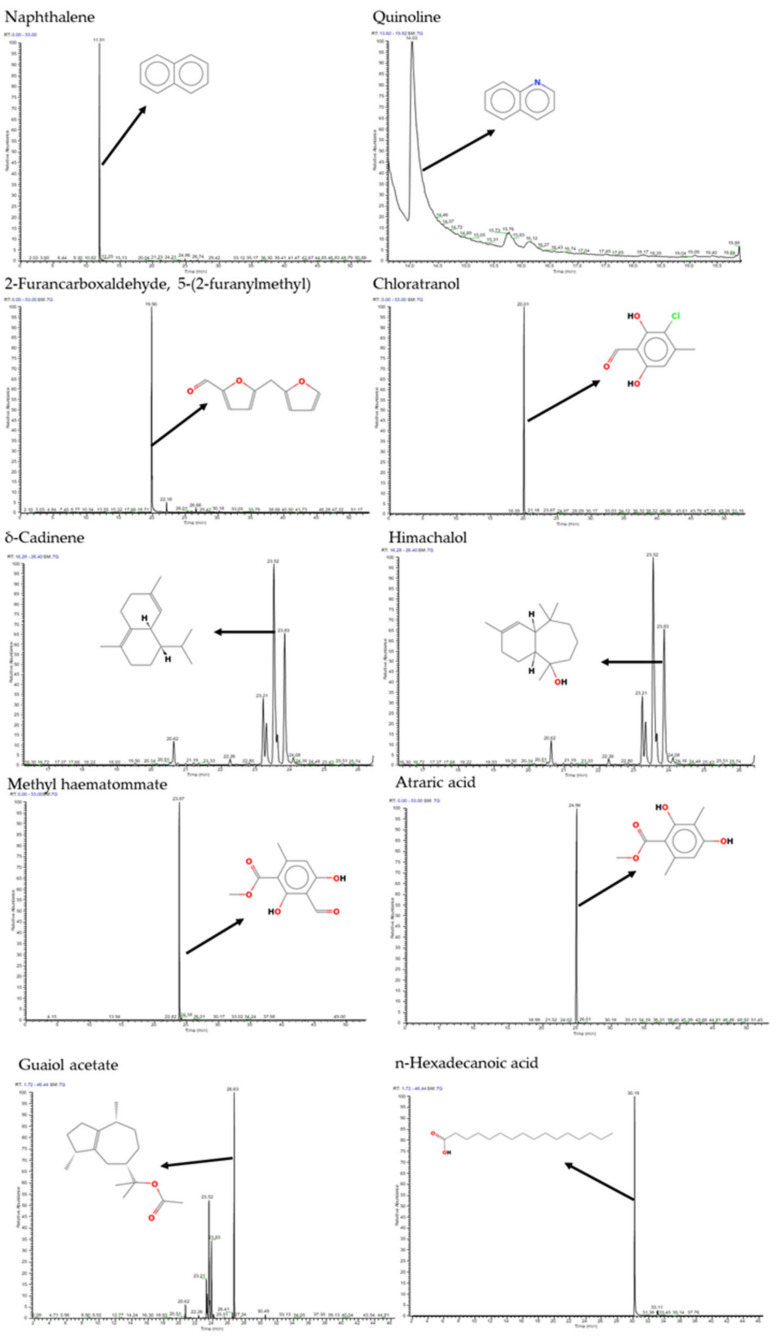
Chromatograms of the main compounds present in PFVC1 obtained by GC-MS.

**Figure 2:**
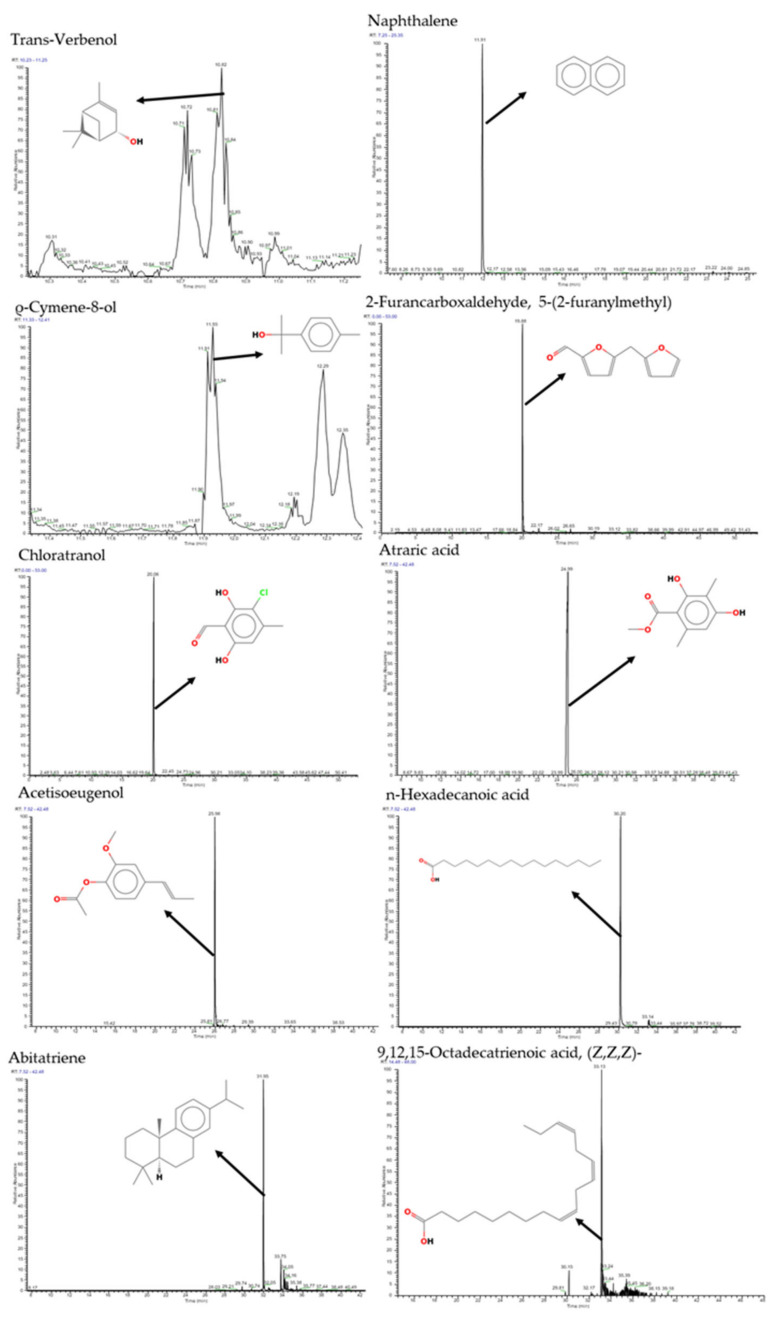
Chromatograms of the main compounds present in PFVC2 obtained by GC-MS.

A total of 15 volatile compounds were detected in PFVC1 and PFVC2. Of these, five compounds were found in both samples, and five compounds were unique to each sample (Table 1). Most of the compounds detected are phenolic cyclic compounds. The corresponding chromatograms are presented in Figure 1 and Figure 2.

The primary compound in both samples was identified as atraric acid, comprising 73.53% and 56.95% of PFVC1 and PFVC2, respectively. Chloroatranol followed as the second most abundant compound, accounting for 19.80% and 24.38% of PFVC1 and PFVC2, respectively. Notably, atraric acid has been previously reported in acetone fractions of methanol extracts of lichens from the Parmeliaceae family, with varying relative abundances [43]. However, our extracts exhibited relatively higher proportions of atraric acid than in the existing literature.

Chloroatranol is derived from the degradation of chloroatranorin, commonly found in oakmoss and treemoss absolutes [12,44]. This compound is a well-known allergen and sensitizer in cosmetic products [45,46,47].

The remaining compounds detected in both extracts showed relative abundances of less than 3.77%. In PFVC1, δ-cadinene, naphthalene, and n-hexadecanoic acid were among the most abundant minor compounds, while in PFVC2, acetisoeugenol, ρ-cymene-8-ol, 2-furancarboxaldehyde, 5-(2-furanylmethyl)-, n-hexadecanoic acid, trans-verbenol, naphthalene, and abietatriene predominated.

Among the minor compounds detected, methyl haematommate has also been identified in volatile fractions of lichens of the Parmeliaceae family [43], whereas n-hexadecanoic acid and naphthalene have previously been reported in volatile compounds from *Lichina pygmaea* [35]. Moreover, abietatriene was reported in essential oils of *Evernia prunastri* and *Evernia divaricata* from Turkey [48], thus further corroborating the presence of these compounds in our samples.

In contrast, a total of eight compounds are reported here for the first time in lichens. Those compounds are as follows: trans-verbenol; ρ-cymene-8-ol; quinoline; 2-furancarboxaldehyde, 5-(2-furanylmethyl)-; δ-cadinene; himachalol; acetisoeugenol; and guaiol acetate. The novelty of these compounds may be attributed to the fact that this is the first report of volatile compounds from the lichen *P. furfuracea* L. (Zopf.) var. *furfuracea* from two different biotopes in Morocco using this novel extraction procedure.

This analysis sheds light on the chemical composition of PFVC1 and PFVC2, highlighting the relatively high abundance of atraric acid and chloroatranol, as well as the presence of novel minor compounds that have never been reported previously in lichens.

### 3.2. Antimicrobial Activity

*Pseudevernia furfuracea* volatile compounds were evaluated for their antimicrobial potential both qualitatively, using the disc diffusion method, and quantitatively, using the well microdilution assay.

Based on the observed diameters of inhibition zones (Table 2), PFVC1 and PFVC2 exhibited antimicrobial activity against all tested microorganisms, with inhibition zone diameters (IZDs) ranging from 6.63 to 40 mm. Notably, the largest IZDs were observed against *P. anguiliseptica*, with values of 36 and 40 mm for PFVC1 and PFVC2, respectively. Similarly, the second most susceptible strain in the solid medium to PFVC1 and PFVC2 was *C. albicans*, with IZD values of 23 and 32 mm, respectively. Conversely, the lowest activity in the solid medium was observed for PFVC1 against *S. typhimurium* and *E. coli* ATCC 25922, with IZDs of 6.63 ± 0.15 mm and 6.73 ± 0.15 mm, respectively.

Quantitatively, the MIC of the volatile compounds of *P. furfuracea* was determined, revealing high antimicrobial activity, with MIC values equal to or below 1000 µg/mL (Table 2). Notably, the most promising activity was observed against *C. albicans*, with MIC values of 62.5 and 31.25 µg/mL for PFVC1 and PFVC2, respectively. Similarly, promising activity was observed against *E. tarda*, with an MIC value of 125 µg/mL for both PFVC1 and PFVC2. In contrast, the highest MIC was observed for *K. pneumoniae*, with a value of 1000 µg/mL for PFVC2. Furthermore, no difference in MIC was observed between the two strains of *E. coli* and *S. aureus* tested in this study.

PFVC1 and PFVC2 exhibited the same MIC values against *E. coli* E1, *S. aureus* S1, *S. aureus* ATCC 29213, *S. typhimurium*, *E. coli* ATCC 29213, *P. anguiliseptica*, *E. tarda*, and *Y. ruckeri*. PFVC1 had a lower MIC value than PFVC2 against *A. baumannii*, *K. pneumoniae*, and *L. anguilarum*, whereas PFVC2 exhibited a lower MIC value than PFVC1 against *B. subtilis*, *C. albicans*, and *A. hydrophila*. Given that the major compounds of both PFVC1 and PFVC2 are the same, the difference in the observed MIC values is likely due to variations in the composition of minor compounds, or due to variations in the abundance of the major compounds.

## 4. Discussion

This work evaluates for the first time the composition and antimicrobial potential of the volatile compounds of the lichen *P. furfuracea* (L.) Zopf. var. *furfuracea* from two different biotopes in Morocco. To our knowledge, very few reports exist about volatile compounds from lichens, due to various reasons, such as the low volatility of lichenic compounds and the low yields of those compounds [49].

Our findings indicate that the major compound in PFVC1 and PFVC2 is atraric acid (73.53 and 56.95%, respectively), followed by chloroatranol (19.80 and 24.38%, respectively). Moreover, eight compounds are reported here for the first time in lichens. Despite the similarities in major compounds between PFVC1 and PFVC2, specifically atraric acid and chloroatranol, which account for 93.33 and 81.33% of their compositions respectively, there are differences in the proportions of these major compounds and the composition of minor compounds. These differences may be due to variations in environmental conditions, the substrate, and altitude between the lichens. Indeed, variation in environmental conditions can be responsible for variation in gene expression [50]. Moreover, variation in the substrate (tree species) can have an impact on the mineral content of lichens [51]. PFVC1 is derived from a lichen growing in a sub-humid region on *Quercus rotundifolia* bark at an altitude of 1644 m, while PFVC2 comes from a lichen developing in a humid region on *Cedrus atlantica* bark at an altitude of 1776 m [52]. Aoussar et al. [15,16] investigated the composition and antimicrobial potential of methanol and acetone extracts of *P. furfuracea* (L.) Zopf. from Khenifra, Morocco. These extracts contained physodalic acid, atranorin, and chloroatranorin. This highlights the unique composition of PFVC1 and PFVC2, which contain atraric acid, a compound from Moroccan *P. furfuracea* described here for the first time.

PFVC1 and PFVC2 exhibited strong antimicrobial activity against human and fish pathogens, with MIC values as low as 32.5 µg/mL against *C. albicans* and no higher than 1000 µg/mL against the multidrug-resistant clinical isolate *K. pneumoniae*. A very interesting result was the high antimicrobial activity against the fish pathogenic bacteria *A. hydrophila*, *E. tarda*, *Y. ruckeri*, and *P. anguiliseptica*. These findings are in opposition with the results of Tas et al. [53], which showed that methanol, acetone, and water extracts from Turkish *P. furfuracea* did not exhibit any antimicrobial potential in a solid medium against *A. hydrophila*, *Aeromonas salmonicida*, *Streptococcus agalactiae*, *Y. ruckeri*, *Enterococcus faecalis*, and *Lactococcus garvieae.* Therefore, this is the first report of the antimicrobial potential of compounds from *P. furfuracea* against fish pathogenic bacteria, highlighting at the same time the potential of volatile compounds from *P. furfuracea* as a source of antimicrobial compounds with a large spectrum of action, covering Gram-positive and Gram-negative bacteria as well as yeast. In addition, PFVC1 and PFVC2 exhibited similar MIC values for antibiotic-resistant and reference strains of *E. coli* and *S. aureus*, suggesting a mechanism of action independent of bacterial resistance pathways.

The antimicrobial potential of PFVC1 and PFVC2 can be partly attributed to their composition of shared and unique bioactive compounds. Atraric acid, the major compound shared between PFVC1 and PFVC2, has shown mixed results regarding its antimicrobial efficacy. While it exhibited no activity against *Porphyromonas gingivalis* and *Streptococcus mutans* at concentrations up to 80 µM when isolated from *Stereocolon paschale* [54], atraric acid from oakmoss absolute displayed strong antimicrobial effects against several Legionella strains, with MIC values ranging from 12 to 26.7 µg/mL across strains including *L. pneumophila*, *L. bozemanii*, and *L. longbeachae* [55].

Both PFVC1 and PFVC2 also contained chloroatranol and n-hexadecanoic acid, which contribute to their overall antimicrobial profile. Chloroatranol, isolated from Turkish *P. furfuracea*, demonstrated activity against a range of pathogens, including *A. hydrophila*, *Bacillus cereus*, *Bacillus subtilis*, *Listeria monocytogenes*, *Proteus vulgaris*, *S. aureus*, *Yersinia enterocolitica*, *C. albicans*, and *Candida glabrata*, with MIC values between 7.7 and 30.6 mM [56]. Additionally, n-hexadecanoic acid showed antibacterial activity against *S. aureus*, *B. subtilis*, *E. coli*, and *K. pneumoniae*, with MIC values ranging from 2.65 to 6.87 µg/mL [57], and was active against *Mycobacterium smegmatis*, a tuberculosis model, with an MIC of 128 µg/mL [58].

Among the compounds unique to PFVC1, δ-cadinene and himachalol exhibited distinct antimicrobial potential. δ-Cadinene, a volatile compound also found in green tea, showed activity against *B. subtilis*, *Bacillus ammoniagenes*, *Streptococcus mutans*, and *Propionibacterium acnes*, with MIC values between 3.13 and 800 µg/mL, though it was less effective against pathogens like *S. aureus* and *Pseudomonas aeruginosa* [59]. Himachalol has shown antifungal activity with an MIC of 250 µg/mL against *Aspergillus fumigatus* and has demonstrated a 60% survival rate in treated mice when administered at 200 mg/kg [60]. Methyl haematommate, another compound specific to PFVC1, has also shown limited antimicrobial potential against *S. mutans* and *P. gingivalis* [54].

Finally, PFVC2 contains 9,12,15-Octadecatrienoic acid, (Z,Z,Z)-, which contributes further to its antimicrobial potential. 9,12,15-Octadecatrienoic acid, (Z,Z,Z)- has shown activity against Gram-positive bacteria such as *Bacillus cereus* and *S. aureus*, with MIC values of 20 and 50 µg/mL, respectively, though it was inactive against Gram-negative bacteria at concentrations up to 1000 µg/mL [61]. This compound also enhanced the efficacy of rifampicin against *M. smegmatis*, reducing its MIC by a factor of four, suggesting a potential synergistic effect [58].

Nevertheless, it is important to note that antimicrobial potential was evaluated for a mixture of compounds and not for isolated pure compounds. Therefore, the observed antimicrobial activities cannot be definitively attributed to any single compound, even to those present in high abundance. Consequently, we cannot exclude the possibility that the antimicrobial activity observed is the result of a synergistic interaction between the various constituents of the volatile compounds of *P. furfuracea*. Thus, a fractionation of the various components of PFVC1 and PFVC2 to obtain pure compounds and the evaluation of the antimicrobial potential of those pure compounds is needed to assess if there could be new candidates for antibiotic applications for human or animal use.

## 5. Conclusions

This study represents the first report on the chemical composition and antimicrobial potential of volatile compounds from *P. furfuracea* var. *furfuracea* from two different biotopes in Morocco. Major constituents identified in both volatile compounds were consistent, predominantly atraric acid and chloroatranol, while differences in minor compounds were observed. Atraric acid was reported for the first time in *P. furfuracea* from Morocco, and eight compounds were detected for the first time in lichens. The antimicrobial activity of volatile compounds was assessed using the disc diffusion and broth microdilution assays, against clinical antibiotic-resistant strains, reference strains, and fish pathogenic strains. This work constitutes the first report of the antimicrobial potential of *P. furfuracea* against fish pathogenic strains. Notably, the most promising activity against fish pathogens was observed in a liquid medium against *A. hydrophila* and *E. tarda*. In addition, potent activity was observed against *C. albicans* in a liquid medium. No significant difference was noted between the antimicrobial potential of PFVCs against antibiotic-resistant and reference strains of *E. coli* and *S. aureus*. However, further investigations are warranted to identify the specific compounds responsible for this antimicrobial potential. Such insights could facilitate the development of novel antimicrobial agents with broad-spectrum activity and potential clinical applications.

## Figures and Tables

**Table 1:** Chemical composition of the volatile compounds from *Pseudevernia furfuracea* (L.) Zopf from two different locations in Morocco obtained by GC-MS. PFVC1, *Pseudevernia furfuracea* volatile compounds from the High Atlas region. PFVC2, *Pseudevernia furfuracea* volatile compounds from the Middle Atlas region.

					Relative Abundance (%)	
Name	Formula	*m*/*z*	Retention Time (min)	Characterized *m*/*z* Fragmentation	PFVC1	PFVC2
Trans-Verbenol	C_10_H_16_O	150.10434	10.819	137.09605; 109.0679; 67.05420	-	2.25
Naphthalene	C_10_H_8_	128.06248	11.906	128.06195; 127.05422	1.23	2.2
ρ-Cymene-8-ol	C_10_H_14_O	150.10434	11.929	135.08034; 132.09329	-	3.04
Quinoline	C_9_H_7_N	129.05771	14.070	129.05716; 128.04941	0.25	-
2-Furancarboxaldehyde, 5-(2-furanylmethyl)-	C_10_H_8_O_3_	176.04712	19.878	110.05623; 53.04685	0.89	2.88
Chloroatranol	C_8_H_7_ClO_3_	186.00767	20.039	184.99997; 140.00228	19.80	24.38
δ-Cadinene	C_15_H_24_	204.18764	23.527	161.13234; 133.10115	1.41	-
Himachalol	C_15_H_26_O	204.18767	23.825	204.18712; 150.03104; 119.08552	0.27	-
Methyl haematommate	C_10_H_10_O_5_	210.05272	24.176	210.05218; 178.025878; 150.03105	0.87	-
Atraric acid	C_10_H_12_O_4_	196.0733	24.978	196.07274; 177.03619; 164.05173	73.53	56.95
Acetisoeugenol	C_12_H_14_O_3_	238.08397	25.993	164.04664	-	3.77
Guaiol acetate	C_17_H_28_O_2_	204.18764	26.634	161.13238; 105.06987	0.82	-
n-Hexadecanoic acid	C_16_H_32_O_2_	256.24017	30.200	73.02841	0.94	2.67
Abietatriene	C_20_H_30_	270.23474	31.946	255.21075; 173.13241	-	1.62
9,12,15-Octadecatrienoic acid, (Z,Z,Z)-	C_18_H_30_O_2_	278.22421	33.130	79.05423; 55.05425	-	0.18
Total					100	100

**Table 2:** Antimicrobial activity of volatile compounds from *Pseudevernia furfuracea* (L.) Zopf. from two different locations in Morocco. IZD, inhibition zone diameter. MIC, minimum inhibitory concentration. PFVC1, *Pseudevernia furfuracea* volatile compounds from the High Atlas region. PFVC2, *Pseudevernia furfuracea* volatile compounds from the Middle Atlas region.

Microorganism	IZD		MIC (µg/mL)	
	PFVC1	PFVC2	PFVC1	PFVC2
*Acinetobacter baumannii* (A1)			125	250
*Escherichia coli* (E1)			500	500
*Klebsiella pneumoniae* (K1)			500	1000
*Staphylococcus aureus* (S1)			250	250
*Staphylococcus aureus* (ATCC 29213)			250	250
*Bacillus subtilis* (ATCC 6633)			500	250
*Salmonella typhimurium* (ATCC 25241)			500	500
*Escherichia coli* (ATCC 25922)			500	500
*Candida albicans* (ATCC 10231)			62.5	31.25
*Aeromonas hydrophila* (DSM 30187)			250	125
*Pseudomonas anguiliseptica* (DSM 12111)			250	250
*Edwardsiella tarda* (DSM 30052)			125	125
*Listonella anguillarum* (ATCC 19264)			250	500
*Yersinia ruckeri* (ATCC 29473)			250	250

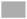
: No halo; 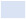: <10 mm; 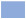: 10–20 mm; 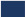: >20 mm.

## Data Availability

The original contributions presented in the study are included in the article/Supplementary Material, further inquiries can be directed to the corresponding author.

## Supplementary Materials

The following supporting information can be downloaded at https://www.mdpi.com/article/10.3390/microorganisms12112336/s1, Figure S1: Pictures of *Pseudevernia furfuracea* (L.) Zopf on *Quercus rotundifolia* L. Table S1: Culture conditions of the strains tested in the antimicrobial assays; Table S2: Resistance profile of the clinical isolates tested in this study following the guidelines recommended by the Antibiogram Committee of the French Microbiology Society [33] and the European Committee on Antimicrobial Susceptibility Testing [34]. R, indicates resistance to the tested antibiotic; S, indicates susceptibility to the tested antibiotic; ‘-’, indicates that the antibiotic was not tested against the strain. Table S3: Inhibition zone diameters obtained with the controls of the disc diffusion assay. ‘-’ not tested.
