# Supplementary material for: In Vitro Antimicrobial Activity of Volatile Compounds from the Lichen Pseudevernia furfuracea (L.) Zopf. Against Multidrug-Resistant Bacteria and Fish Pathogens
Source: Microorganisms. 2024 Nov 15;12(11):2336. doi: 10.3390/microorganisms12112336 (PMC11596387; doi:10.3390/microorganisms12112336)
Supplement: Supplementary file 1 [file microorganisms-12-02336-s001.zip › microorganisms-3249216-supplementary.pdf]

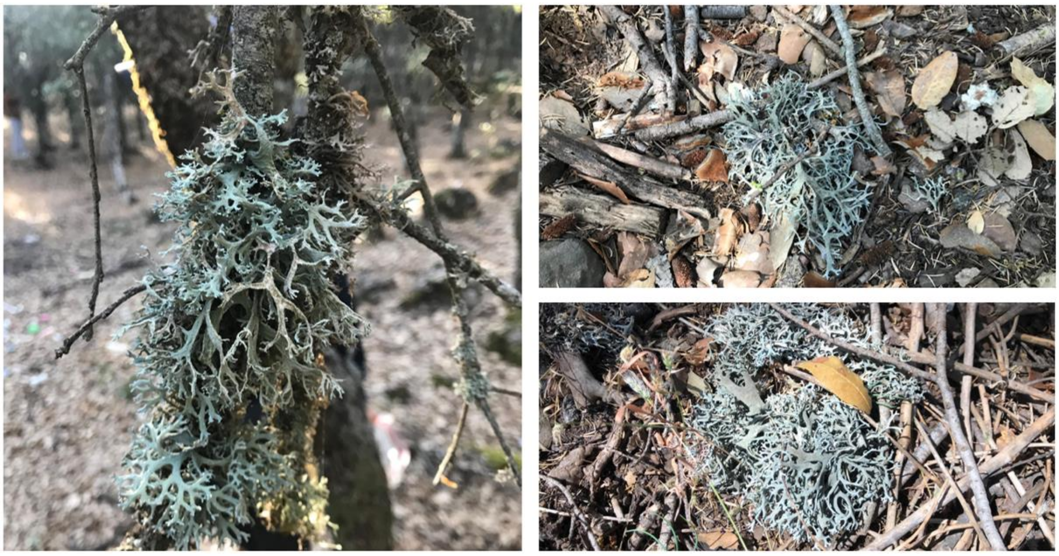

Figure S1. Pictures of *Pseudevernia furfuracea* (L.) Zopf on *Quercus rotundifolia* L.

Table S1. Culture conditions of the strains tested in the antimicrobial assays.

Strain	Code	Culture medium	Incubation temperature	Incubation time
Aeromonas hydrophila	DSM 30187	Tryptic soy agar	28°C	24h
Pseudomonas anguiliseptica	DSM 12111	Tryptic soy agar	25°C	48-72h
Edwardsiella tarda	DSM 30052	Tryptic soy agar	28°C	24h
Listonella anguillarum	ATCC 19264	Tryptic soy agar	30°C	48h
Yersinia ruckeri	ATCC 29473	Tryptic soy agar	30°C	48h
Escherichia coli	ATCC 25922	Mueller-Hinton agar	37°C	24h
Escherichia coli	E1	Mueller-Hinton agar	37°C	24h
Bacillus subtilis	ATCC 6633	Mueller-Hinton agar	37°C	24h
Staphylococcus aureus	ATCC 29213	Mueller-Hinton agar	37°C	24h
Staphylococcus aureus	S1	Mueller-Hinton agar	37°C	24h
Salmonella typhimurium	ATCC 25241	Mueller-Hinton agar	37°C	24h
Klebsiella pneumoniae	K1	Mueller-Hinton agar	37°C	24h
Acinetobacter baumanii	A1	Mueller-Hinton agar	37°C	24h
Candida albicans	ATCC 10231	Sabouraud-Dextrose agar	37°C	48h

Table S2. Resistance profile of the clinical isolates tested in this study following the Antibiogram Committee of the French Microbiology Society [33] and the European Committee on Antimicrobial Susceptibility Testing [34]. R, indicates resistance to the tested antibiotic; S, indicates susceptibility to the tested antibiotic; '-', indicates that the antibiotic was not tested against the strain.

Antibiotic		*Klebsiella pneumoniae* K1	*Escherichia coli* E1	*Acinetobacter baumanii* A1	*Staphylococcus aureus* S1
Amoxicillin	25 µg	R	R	-	-
Ticarcillin	75 µg	R	R	R	R
Ticarcillin + clavulanic acid	75/10 µg	-	-	R	-
Ceftazidime	30 µg	R	R	R	-
Amoxicillin + clavulanic acid	25/10 µg	R	R	R	-
Oxacillin	5 µg	-	-	-	R
Minocycline	30 UI	-	-	-	S
Vancomycin	30 µg	-	-	-	S
Ceftriaxone	30 µg	R	R	R	-
Aztreonam	30 µg	R	R	R	-
Teicoplanin	30 µg	-	-	-	S
Cefoxitin	10 µg	R	R	R	R
Cefalotin	30 µg	S	R	R	-
Pefloxacin	5 µg	S	R	R	-
Kanamycin	30 µg	-	-	-	R
Erythromycin	30 µg	-	-	-	R
Netilmicin	30 µg	S	S	R	-
Amikacin	30 µg	S	S	R	-
Imipenem	10 µg	R	R	R	R
Sulfamethoxazole-Trimethoprim	23,75 µg/1,25 µg	S	S	R	R
Cefotaxime	30 µg	S	R	R	R
Norfloxacin	5 µg	R	S	R	R
Moxifloxacin	5 µg	R	S	-	-
Piperacillin + tazobactam	75/10 µg	-	-	R	-
Chloramphenicol	30 µg	-	-	R	-
Fusidic acid	10 µg	-	-	-	R
Streptogramins	15 µg	-	-	-	S
Penicillin G	6 µg	-	-	-	R

Table S3. Inhibition zone diameters obtained with the controls of the disc diffusion assay. '-' not tested.

Strain	Code	DMSO	Enrofloxacin (1 mg/ml)	Nystatin (1 mg/ml)
Staphylococcus aureus	ATCC 29213	0 ± 0	37 ± 0	-
Bacillus subtilis	ATCC 6633	0 ± 0	43.5 ± 0	-
Salmonella typhimurium	ATCC 25241	0 ± 0	44 ± 0	-
Escherichia coli	ATCC 25922	0 ± 0	43.5 ± 0	-
Aeromonas hydrophila	DSM 3018	0 ± 0	48 ± 0	-
Pseudomonas anguiliseptica	DSM 12111	0 ± 0	75 ± 0	-
Candida albicans	ATCC 10231	0 ± 0	-	26.67 ± 0.58
Edwardsiella tarda	DSM 30052	0 ± 0	50.5 ± 0.71	-
Listonella anguillarum	ATCC 19264	0 ± 0	0 ± 0	-
Yersinia ruckeri	ATCC 29473	0 ± 0	53 ± 0	-
Klebsiella pneumoniae	K1	0 ± 0	-	-
Escherichia coli	E1	0 ± 0	-	-
Acinetobacter baumanii	A1	0 ± 0	-	-
Staphylococcus aureus	S1	0 ± 0	-	-
